# Relationship between the Onset of Depression and Stress Response Measured by the Brief Job Stress Questionnaire among Japanese Employees: A Cohort Study

**DOI:** 10.1371/journal.pone.0056319

**Published:** 2013-02-12

**Authors:** Keiko Wada, Toshimi Sairenchi, Yasuo Haruyama, Hiromi Taneichi, Yumiko Ishikawa, Takashi Muto

**Affiliations:** 1 Department of Public Health, Dokkyo Medical University School of Medicine, Shimotsugagun-Mibu, Tochigi, Japan; 2 Dokkyo Medical University School of Nursing, Shimotsugagun-Mibu, Tochigi, Japan; The University of Hong Kong, Hong Kong

## Abstract

**Background:**

The proportion of Japanese workers experiencing intense worry or stress during working life is in excess of 60%, and the incidence of psychiatric disorders and suicide due to psychological burden from work duties is increasing. To confirm whether the stress response measured by the Brief Job Stress Questionnaire (BJSQ) can identify risk for depression, a cohort study was conducted to evaluate whether the stress response measured by BJSQ was associated with the onset of depression.

**Methods:**

A total of 1,810 participants aged 20–70 years in 2005 completed the stress response of the BJSQ and were followed-up until August, 2007 by examining sick pay records. Depression was defined by a description in sick pay records that included “depression” or “depressive symptoms” as a reason for sick leave according to a physician's medical certificate. The participants were divided into quartiles (Ql, Q2, Q3, and Q4) according to the total stress response score of BJSQ at baseline. Furthermore, the participants were divided into a higher score category (Q4) and a lower score category (Q1–Q3). Risk ratios of the stress response of the BJSQ for onset of depression were calculated using a multivariable Cox proportional hazard model.

**Results:**

Among 1,810 participants, 14 developed depression during a mean of 1.8 years of follow-up. The risk ratio was 2.96 (95% confidence interval [CI], 1.04–8.42, p for trend = 0.002) when the higher stress response score category of BJSQ was compared with the low stress response score category for sick leave due to depression. After adjusting for gender, age, marital status, and having children, the risk ratios were similar to no adjustment.

**Conclusions:**

These findings suggest that the stress response measured by the BJSQ can demonstrate risk for the onset of depression.

## Introduction

Depression is a common mental illness and a leading cause of disability worldwide, with an estimated 350 million people affected [1]. In Japan, the morbidity related to psychiatric and behavioral disorders was 232/100,000 in 2008 [2], and there is a continued increasing trend [3]. According to national statistics, the number of suicides exceeded 30,000 over a 12-year period starting from 1998. Of these suicides, 30% were employees and salaried workers who had psychological burden related to work duties. Over 60% of workers in Japan reported experiencing intense worry or stress related to employment [3], and in 2008 over 50% of responding enterprises reported that “workers increasingly dealt with mental health problems” [4]. Therefore, mental health care for employees and mental health measures to address these trends are an important issue at worksites.

“Guidelines for Promoting Mental Health Care in Workplace Enterprises” developed by the Japan Ministry of Health, Labour and Welfare (JMHLW), were issued for Japanese workplaces in 2000 [5]. In 2006, these guidelines were reviewed and revised as the “Guidelines for Maintenance and Promotion of Mental Health for Workers”. Currently, mental and physical health preservation and promotion for workers are addressed by mental health provisions utilizing the following resources: 1) workers themselves, 2) co-workers, 3) on-site occupational health workers, and 4) non-workplace resources [6].

In 1998, the Brief Job Stress Questionnaire (BJSQ) was developed by a research group commissioned by JMHLW [7]. The BJSQ is a simplified questionnaire for employees with a relatively small number of question items. It is a 57-item multidimensional job stress questionnaire composed of questions related to job stressors (17 items), stress response (29 items), social factors (9 items), and work and life satisfaction (2 items). Reliability and validity of the BJSQ among Japanese employees has been established [7]. Subsequent diverse studies have reported the association between high job stresses measured by the BJSQ and long work hours [8], overtime work [9], and shift work [10]. Moreover, the level of job stress and stress response for different occupational categories was assessed. BJSQ-measured job stress has been reported to be associated with high stress response among nurses [11]. The association of BJSQ-measured job stressors and social support with other factors was assessed among physicians [12]. BJSQ was used to measure job stressors, stress response and social support among teachers [13]. The differences in BJSQ-measured job stressors and social support during a traumatic event experience for firefighters were clarified [14]. A significant relationship between BJSQ-measured job stressors, stress response and social support among overseas cooperation volunteers and office workers was found [15], [16]. These studies were conducted in a cross-sectional design. In addition, some interventional studies, such as those by Kobayashi et al., (2008) Hase et al., (2008) and Ikegami et al. (2008), have shown that mental health promotional programs could reduce workers' job stress and stress response [17]–[19]. Kawagami et al., (2006) and Mineyama et al. (2007) demonstrated the effectiveness of supervisor training using the BJSQ [20], [21]. Therefore, the BJSQ has proven to be a useful tool for mental health care at Japanese worksites.

Although no cohort study has been conducted using the BJSQ, there has been some research on the prediction of onset of major depression related to work stress [22]–[24], problematic interpersonal relationships [25], and stressful events [26]. Among 2,821 Belgian workers, job stress assessed by the Job Content Questionnaire increased the risk of developing high levels of depression symptoms assessed by the Iowa form of the Center for Epidemiological Studies-Depression Scale after a mean follow-up time of 6.6 years [27]. The General Health Questionnaire (GHQ) was used in a two-year cohort study conducted on 462 Japanese workers the findings of which showed that both job strain and job demands were associated with poor mental health [28]. The self-administered Center for Epidemiologic Studies Depression (CES-D) Scale questionnaire was used to assess 11,552 French workers. The psychosocial work environment characteristics, psychological job demands, latitude in decision-making and social support at work were found to be significant predictors of subsequent depressive symptoms [29]. However, it remains unclear whether the BJSQ can predict future onset of depression with consideration of temporality.

It is important to determine whether stress questionnaires in the workplace do more than simply assess current stress states. An assessment method for depression of high risk individuals is needed. The aim of the present study was to evaluate whether the stress response measured by BJSQ was associated with the future onset of depression.

## Methods

### Study Cohort

**Figure 1:**
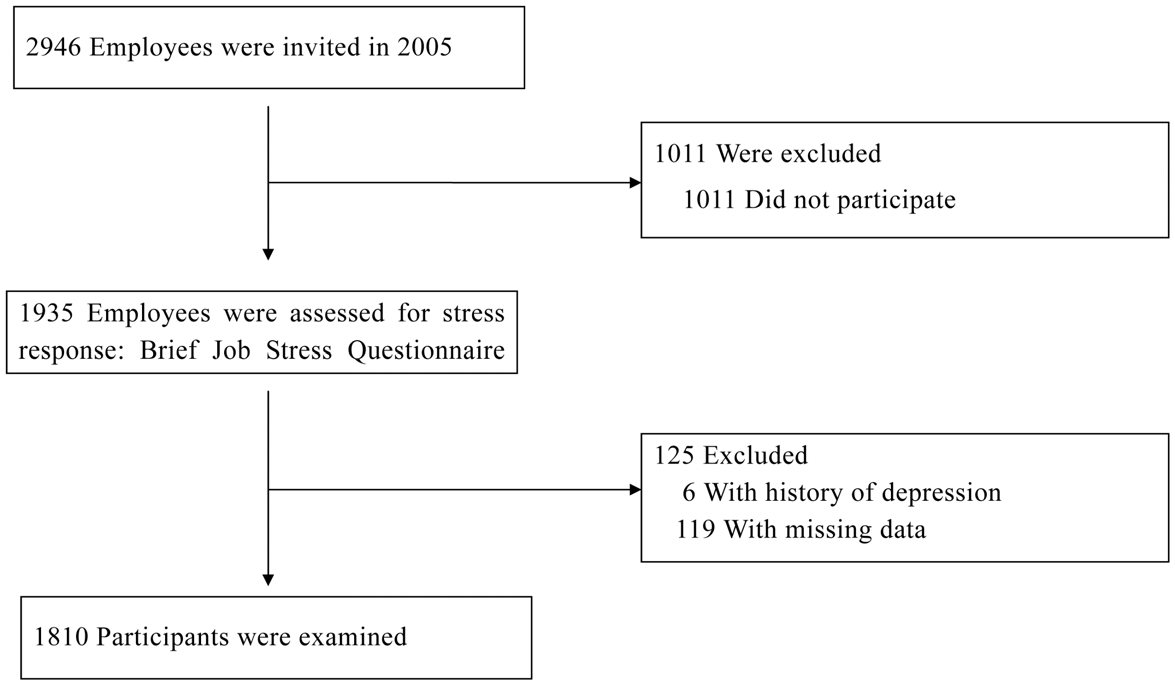
Detailed flow diagram of the study participants.

In the present cohort study, 2,946 employees, aged 20–72 years, who worked in a software development company were invited to complete a mental health questionnaire conducted by Healthcare Marketing Intelligence Inc. (Tokyo, Japan) in September 2005. A total of 1,935 employees completed the questionnaire and 1,011 employees did not participate, for a response rate of 65.7%. At baseline, 6 employees with a history of depression (0.3%) which were identified by past sick pay records and 125 employees (6.5%) with missing data were excluded. Thus, 1,810 participants (1,362 men, 448 women; aged 20–70 years) were tracked by sick pay records from 2005 to August, 2007 (Fig. 1). Response group compared with non-response group was younger (35.6 years±8.5 vs. 37.0 years±9.9, p<0.001) and a lower proportion of males (75.3% vs.81.5%, p<0.001).

Anonymous data were received from Healthcare Marketing Intelligence Inc. During data collection, informed consent was not obtained from all participants as responses were anonymous. However, the protocol of this cohort study was approved by the institutional review board of Dokkyo Medical University School of Medicine.

### Assessment of Stress Response

BJSQ, a 57-item multidimensional job stress questionnaire, was used to measure stress response. BJSQ includes: stress related to work (17 items: e.g., quantitative job demands, qualitative job demands, and control), stress response (29 items: e.g., psychological stress response and physical stress response), and social factors (11 items: e. g., social support). The validity and reliability of this questionnaire was previously examined with 2,428 Japanese workers [7]. All the scales on job stress presented acceptable alpha coefficients (i.e., quantitative job demand: 0.66; qualitative job demand: 0.68; support from family and friends: 0.82). Particularly, the scales on job stress response presented high alpha coefficients reflecting high internal consistency (i.e., psychological stress response: 0.85; physical stress response: 0.79).

In another study with 1,084 Japanese workers, the scales on job stress presented high alpha coefficients (i.e., job demand: 0.70; support from superiors and colleagues: 0.79; psychological stress response: 0.88; physical stress response: 0.73) [15].

Stress response is a subscale evaluated by 29 items of the BJSQ. The total stress response consisted of 18 items related to psychological stress response including such manifestations as lassitude, irritation, fatigue, anxiety, and depression, and 11 items which related physical stress response to somatic symptoms. In the present analysis, the total score of the 29-item stress response, 18-item psychological stress response, and 11-item physical stress response were used. The score was calculated for each item and response options were based on a 4-point Likert scale ranging from “strongly disagree” = 1 to “strongly agree” = 4. Approximately ten minutes were required to answer all items. Higher scores were related to higher stress response from the employee, so four quartiles were calculated using the total scores of stress response of BJSQ.

### Incident of Depression

The participants were followed-up during a mean of 1.8 years by examining sick leave records with medical certificates. Sick pay is paid to the employee by health insurance in Japan and sick leave is defined as leave of three or more days due to employee illness. A medical certificate issued by a physician is required for application for sick pay. In the present study, the sick pay records with medical certificates were linked to the mental health questionnaires by insurance numbers of the employees. Depression was defined by a description of “depression” or “depressive symptoms” as a reason for sick leave according to the medical certificates. The day of incident of depression was defined as the first day of the sick leave.

### Statistical Analysis

The participants were divided into four quartiles (Q1, Q2, Q3, and Q4) according to the total score in stress response of the BJSQ at baseline (Table 1). Some lower quartiles included no subjects with sick leave. Therefore, the participants were divided into a higher score category (Q4) and a lower score category (Q1–Q3) (Table 2). The participants were also divided according to each subscale (psychological stress response and physical stress response). The person-month was calculated by the follow-up period. Risk ratios of the total stress response of the BJSQ and the subscales for onset of depression were calculated using a Cox proportional hazard model. A crude risk ratio was included in Model 1. The analysis was repeated with age (years) (Model 2), married (yes or no) (Model 3), and has children (Model 4) as covariates in addition to gender. The statistical analyses were conducted using SAS, version 9.1 (SAS Institute, Inc., Cary, NC, USA).

**Table 1:** Baseline characteristics of the study participants among 1810 Japanese employees in 2005.

	total	Q1	Q2	Q3	Q4	
Number of participants	1810	446	475	422	467	
Male, n (%)	1362 (75.2)	337 (75.4)	377 (79.6)	309 (73.5)	339 (72.3)	0.070
Age, mean±SD	35.6±8.5	35.8±8.9	35.8±8.7	36.1±8.6	34.8±7.8	0.081
Work hours per day, mean±SD	9.6±5.2	9.5±5.7	9.8±6.9	9.5±4.4	9.7±2.7	0.651
Administrative post, n (%)	767 (42.4)	192 (43.0)	197 (41.5)	184 (43.6)	194 (41.5)	0.889
Marriage, n (%)	905 (50.0)	246 (55.2)	244 (51.4)	209 (49.5)	206 (44.1)	0.009
Children, n (%)	665 (36.7)	176 (39.5)	189 (39.8)	156 (37.0)	144 (30.8)	0.016

Tested by ANOVA for age and work hours per day, and by χ^2^ test for gender, administrative post, marriage and children.

BJSQ: Brief Job Stress Questionnaire.

**Table 2:** Sick leave due to depression by scores of stress response of BJSQ.

	Score for stress response of BJSQ			
	Q1	Q2	Q3	Q4
Total stress response of BJSQ				
No. of participants	446	475	422	467
No. of sick leave related to depression	0	2	5	7
Person -years	820.7	864	759.6	822.2
Sick leave rate per 1,000 person-years	0	2.3	6.6	8.5
Total psychological stress response of BJSQ				
No. of participants	468	403	500	439
No. of sick leave related to depression	0	0	7	7
Person -years	856.3	737.8	903.2	769.2
Sick leave rate per 1,000 person-years	0	0	7.7	9.1
Total physical stress response of BJSQ				
No. of participants	502	387	475	446
No. of sick leave related to depression	1	1	6	6
Person -years	922.8	698.2	865.5	780
Sick leave rate per 1,000 person-years	1.1	1.4	6.9	7.7

BJSQ: Brief Job Stress Questionnaire.

## Results

The baseline characteristics of the study participants according to their stress response on the BJSQ are shown in Table 1. The proportion of participants who were ‘married’ (p for difference = 0.009) and ‘had children’ (p for difference = 0.016) was different significantly. For sex, age, working hours per day and administrative post, no significant differences was observed among the four quartiles according to the stress responses of BJSQ

During the mean follow-up period of 1.8 years, onset of depression was observed in 14 subjects. Table 2 shows the distribution of subjects in quartiles for total, psychological and physical stress response.

Table 3 shows risk ratios of stress response of the BJSQ (high vs. low) and the subscales for onset of depression.

**Table 3:** Risk ratios for sick leave due to depression by scores of stress response of BJSQ.

Score for stress response of BJSQ	No. of participants	Person -years	No. of sick leave related to depression	Sick leave rate per 1,000 person-years	Model 1	Model 2	Model 3	Model 4
Total stress response of BJSQ								
Low (Q1–Q3)	1343	2444.3	7	2.86	1.00	1.00	1.00	1.00
High (Q4)	467	822.2	7	8.51	2.96 (1.04–8.42)	2.94 (1.03–8.40)	2.86 (1.00–8.18)	2.97 (1.04–8.49)
P for trend					0.002	0.002	0.002	0.002
Total psychological stress response of BJSQ								
Low (Q1–Q3)	1371	2497.3	7	2.80	1.00	1.00	1.00	1.00
High (Q4)	439	769.2	7	9.10	3.22 (1.13–9.18)	3.12 (1.09–8.90)	3.01 (1.05–8.64)	3.19 (1.11–9.16)
P for trend					0.001	0.001	0.001	0.001
Total physical stress response of BJSQ								
Low (Q1–Q3)	1364	2486.5	8	3.22	1.00	1.00	1.00	1.00
High (Q4)	446	780.0	6	7.69	2.37 (0.82–6.83)	2.45 (0.85–7.08)	2.40 (0.83–6.92)	2.42 (0.84–6.99)
P for trend					0.036	0.029	0.034	0.032

Model 1: Not adjusted.

Model 2: Adjusted for gender and age.

Model 3: Adjusted for gender and marriage.

Model 4: Adjusted for gender and child.

BJSQ: Brief Job Stress Questionnaire.

P for trend :Analyses scores of stress response as continuous variables.

Model 1 did not adjust for the variables in total stress response of the BJSQ. Compared with the lower stress response category, the risk ratios of the higher stress response category of the BJSQ for sick leave due to depression were 2.96 (95% confidence interval (CI): 1.04 to 8.42, p for trend = 0.002).

For the total physical stress response of the BJSQ, compared with the lower stress response category, the risk ratios of higher physical stress response of the BJSQ for sick leave due to depression were 3.22 (95% CI: 1.13 to 9.18, p for trend = 0.036).

For the total psychological stress response of BJSQ, compared with the lower stress response category, the risk ratios of higher physical stress response of BJSQ for sick leave due to depression were 2.37 (95% CI: 0.82 to 6.83, p for trend = 0.036). After adjusting for gender and age (Model 2), for the total stress response of the BJSQ, the risk ratios of higher stress response category were 2.94 (95% CI: 1.03 to 8.40, p for trend = 0.002).

For the total physical stress response of the BJSQ, the risk ratio of the higher physical stress response of the BJSQ for sick leave due to depression was 2.45 (95% CI: 0.85 to 7.08, p for trend = 0.029).

Model 3 was adjusted for gender and marriage and Model 4 was adjusted for gender and having children, and the results were similar to Model 1 and Model 2.

## Discussion

The present study clarified that high levels of stress response, as measured by the BJSQ, had a significant relationship to the onset of depression among Japanese employees at a software company. The results of the present study should provide the primary evidence to identify and address mental health problems for employees in the workplace using the stress response of BJSQ. There are several strengths for the present research. First, to our knowledge, this is the first study to show the relationship between the BJSQ-measured stress response and the appearance of depression and consider the temporal relationship of onset of depression using a prospective cohort study. Secondly, the stress response measured by the BJSQ shows the relativity of depression and the stress response might forecast the onset of depression according to the stress response demonstrated by the BJSQ and risk of depression. The evidence indicates that it is necessary for occupational health services to pay more attention to employees' mental health and supply mental health programs for employees who have a higher stress response. Thirdly, a relatively large number of subjects were observed in the present study.

Depression is a multifactorial disorder and has various symptoms. Considering the pre-stage of depression, we focused on the stress response as a risk factor for depression. Previous studies showed that worker participation in intervention programs improved stress response and prevented an increase in mental health problems or depression [17], [30]. In those studies, an interventional study design was performed, although those temporal results also support our findings. It is hypothesized that the stress response measured by the 29 items provides a strong likelihood of predicting the onset of depression.

The 29-item stress response (18-items of psychological stress and 11-items of physical stress) measures the symptoms of lassitude, irritation, fatigue, anxiety and depression. These psychological items were developed by using many existing stress-related questionnaires, such as the Center for Epidemiologic Studies Depression Scale (CES-D), Profile of Mood States (POMS), and State-Trait Anxiety Inventory (STAI) [7]. Previous studies reported that CES-D [31], POMS [32], and STAI [33] were related to depression. According to lateral research, the correlation is high between the depression sub-scale of BJSQ and the CES-D [34]–[36]. In another study, the 11-item physical stress response was developed using the somatic symptoms from Screener for Somatoform Disorders (SSD) and WHO-Subjective Well-being Inventory (SUBI) [7]. Some previous studies showed a strong association between somatic symptoms and depression [37]–[39]. In our study, physical stress responses showed no relationship to depression. Possible reasons are that a shorter follow-up period and lower number of depression events attenuated the statistical power of physical stress response. However, the total stress response including the psychological and physical stresses had a significant relationship to depression after adjusting for some confounding factors like gender, age, marriage, and having children. Therefore, the stress response of the BJSQ, including physical response, is considered to be a reliable and valid tool for assessing the onset of depression.

There are several limitations of this research. First, the diagnosis of depression was not made using the Diagnostic and Statistical Manual of Mental Disorders, 4th edition (DSM-IV). However, depression was confirmed by attestation in the present study. The diagnosis of depression might include burnout; however, burnout has been reported as a mediator in the association between job strain and depression [39] and the impact would be minimal. Secondly, although the response rate was 66%, compared to the non-response group, the response group was younger and included more males. Therefore, selection bias could be related to participant self-selection. Thirdly, the participants of this study were workers of one occupational category located in a limited geographic region. Although the results of this research are important, the generalizability is uncertain because all study participants worked in one company. Further studies are warranted to examine other occupational categories and locations. Depression could be chronic or acute and less severe symptoms may be found prior to the actual onset of depression [40]. Therefore, a longer follow-up period in a different cohort may reveal different results. Fourthly, stress response of the BJSQ was used in this study, but other subscales of BJSQ, such as job stressors and social support did not show a relationship to onset of depression. Furthermore, we did not collect data on socioeconomic status and job title or discuss the relationship of these factors with depression.

The percent of depression histories in this study (0.3%) was lower than that in the general population (5%) reported by WHO. There are possible reasons for the low percent of depression histories among the active workers in our study. For example, once a worker has depression there could be a long absence from the job with a difficult recovery [41] or a high recurrence and frequency of sick leave. Also the consultation rate for depression in Japan is about half that of the United States according to previous studies [42]. Japanese workers might be reluctant to speak about a past history of treatment or counseling for mental health problems.

Our results indicate the relationship between the stress reaction and onset of depression. The significance of this research is that the BJSQ stress response might be a reliable assessment tool for depression onset. Further research is needed to test the validity and reliability of the BJSQ as an assessment tool. In conclusion, these findings suggest that the stress response items on the BJSQ are likely to identify risk for depression onset.
